# Modeling the Differentiation of Embryonic Limb Chondroprogenitors by Cell Death and Cell Senescence in High Density Micromass Cultures and Their Regulation by FGF Signaling

**DOI:** 10.3390/cells12010175

**Published:** 2022-12-31

**Authors:** Cristina Duarte-Olivenza, Juan M. Hurle, Juan A. Montero, Carlos I. Lorda-Diez

**Affiliations:** Departamento de Anatomía y Biología Celular and IDIVAL, Universidad de Cantabria, 39011 Santander, Spain

**Keywords:** apoptosis, cell senescence, chondrogenesis

## Abstract

Considering the importance of programmed cell death in the formation of the skeleton during embryonic development, the aim of the present study was to analyze whether regulated cell degeneration also accompanies the differentiation of embryonic limb skeletal progenitors in high-density tridimensional cultures (micromass cultures). Our results show that the formation of primary cartilage nodules in the micromass culture assay involves a patterned process of cell death and cell senescence, complementary to the pattern of chondrogenesis. As occurs in vivo, the degenerative events were preceded by DNA damage detectable by γH2AX immunolabeling and proceeded via apoptosis and cell senescence. Combined treatments of the cultures with growth factors active during limb skeletogenesis, including FGF, BMP, and WNT revealed that FGF signaling modulates the response of progenitors to signaling pathways implicated in cell death. Transcriptional changes induced by FGF treatments suggested that this function is mediated by the positive regulation of the genetic machinery responsible for apoptosis and cell senescence together with hypomethylation of the *Sox9* gene promoter. We propose that FGF signaling exerts a primordial function in the embryonic limb conferring chondroprogenitors with their biological properties.

## 1. Introduction

Cell death is a constant event accompanying the differentiation, growth, and tissue remodeling of biological systems [1]. In embryonic systems, cell death may appear scattered in the differentiating tissues or, most often, it takes place in a massive fashion sculpting the shape and/or the structure of the organ primordia. In adult individuals, cell death is a basic mechanism of tissue turnover, but accumulating evidence indicates that it is also of great importance in stem cell and tumor biology [2].

The formation of the vertebrate skeleton is accompanied by programmed cell death and cell senescence within or around the differentiating skeletal tissues, including the cartilaginous bone primordia and the developing joints [3,4]. The formation of the cartilaginous template that prefigures the future chondral bones is a critical step of skeletogenesis [5]. The regulation of the cellular mass of the cartilage templates to establish their size and morphology might be among the functions of cell death in skeletogenesis [6]. In embryonic tissues, a variable intensity of dead cells is recognizable around the zones of cartilage formation [4,7], but the contribution of cell degeneration to the early steps of skeletogenesis has not received much attention. Remarkably, dysregulation of these dying processes causes skeletal malformations [8].

Genetic and classical embryological studies in vivo have identified complex signaling networks that influence the formation of the skeleton, including WNTs and BMPs, but FGFs appear to exert a central role in the formation of most cartilages [9,10,11]. Most of these signals have dual opposite functions, being growth- and differentiation- or cell death-inducing signals in a stage- and regional- dependent fashion [4,12,13,14,15]. The design of an in vitro model to analyze mechanisms involved in the control of programmed cell death is of great interest to understand this important biological process.

The study of the regulatory signals and the cellular basis of chondrogenesis have been largely addressed in vitro by employing a tridimensional organoid-like culture assay termed the “micromass culture”. This assay mimics in vitro changes associated with skeletogenesis in vivo [16] and has been proposed to replicate the regulatory signals involved in cartilage formation [17]. Studies in monolayer culture provided evidence for the association between apoptosis and chondrodifferentiation [18], but the micromass culture assay has largely been employed in studies focused on the growth and differentiation of progenitors, and less attention was paid to clarify the potential implications of cell death [19].

In this work, we have analyzed the pattern of cell death and the role of FGF signaling in primary micromass cultures of limb skeletogenic progenitors. For this study, we chose the undifferentiated autopod mesodermal cells whose fate in vivo is to form digits or to be eliminated by cell death and senescence in the interdigital regions [4,20]. We show that the formation of primary cartilage nodules in the micromass culture assay involves a patterned process of cell death and cell senescence, complementary to the pattern of chondrogenesis. Treatments with FGF2 alone or in combination with growth factors active during limb skeletogenesis revealed that, in addition to the well-known function of FGF signaling directing the growth of the limb primordia, this pathway modulates the response of progenitors to signals implicated in the regulation of programmed cell death. Transcriptional changes induced by FGF treatments suggest that this function is mediated by positive regulation of the genetic machinery responsible for apoptosis and cell senescence. We propose that FGFs not only direct limb outgrowth but also serve a morphogenetic function tuning the response of progenitors to WNT and BMP signaling.

## 2. Materials and Methods

We employed Rhode Island chicken embryos of 4.5 days for incubation, which represent stage HH25. This stage precedes in two days the establishment of the areas of interdigital cell death.

### 2.1. Micromass Cultures and Treatments

**Figure 1:**
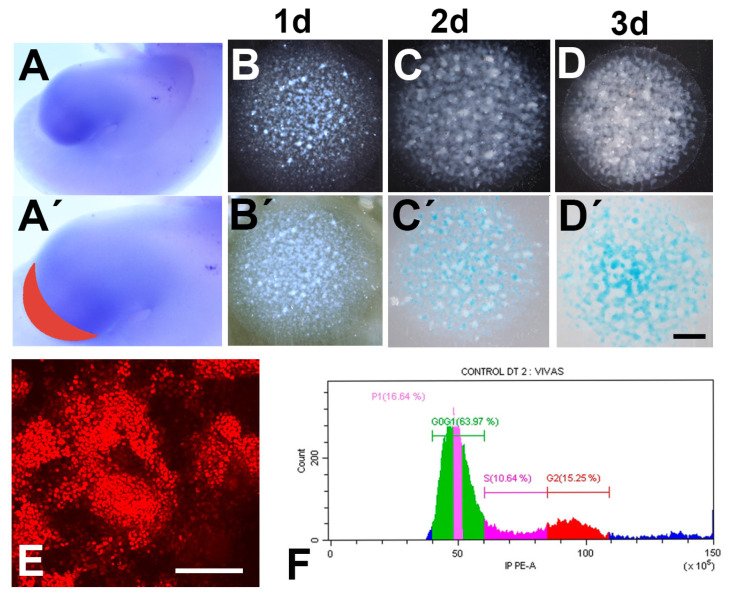
(**A**–**A’**) In situ hybridization of the embryonic leg bud at stage 25, showing the expression of *HoxA13*. **A’** the tissue selected for the experiments is illustrated in red. (**B**–**D**) are dark field low magnification views of micromasses cultured for 24 (**B**), 48 (**C**), and 72 h (**D**). (**B’**–**D’**) illustrate the same cultures after Alcian blue cartilage staining. Note the appearance of Alcian blue-positive nodules on Day 2 of culture. (**E**) 3-day-old micromass section immunolabeled for SOX9. (**F**) Flow cytometry histogram showing the cell cycle of 2 days cultures grown in DMEM. Scale bar in **B**–**D’** = 1 mm; scale bar in E = 100 µm.

The leg buds were excised from the embryo and immersed in L-15 culture medium (Leibovitz medium, Lonza Group) and the undifferentiated tissue of the distal margin of the limb (“progress zone”) expressing *HoxA13* (Figure 1A–A’) was dissected free under the dissecting microscope. To dissociate the cell components, samples were first incubated at 37 °C in 0.25% trypsin (Sigma) for 6 min and next in 0.25% collagenase (Worthington) for 12 min. Digestion was blocked by addition of 1 mL of DMEM (Dulbecco´s modified eagle medium, Lonza Group) with 10% fetal bovine serum (FBS, Lonza Group) where cells were dissociated by gently pipetting. Next, 9 mL of L-15 medium were added to the dissociated tissue and filtered through a 70 μm strainer (Miltenyi Biotec) to remove clumps of undissociated tissue and ectodermal debris. The cell suspension was centrifuged at 1000 rpm for 10 min and re-suspended in DMEM/10% FBS medium. Cell density was adjusted to 3 × 10^5^ cells/mL, and 10 µL drops were pipetted into each well of a 48-well plate (Thermo Scientific), allowed to be attached for 2 h in an incubator (37 °C and 5% CO2) and then 200 µL of the selected growth medium was added into each well. DMEM with or without 10% FBS containing 100 units/mL penicillin and 100 mg/mL streptomycin were employed as culture medium.

Cell death, cell senescence, cell proliferation, DNA methylation, and transcriptional changes, were analyzed in control cultures and experimental cultures treated with recombinant proteins and signaling inhibitors added to the culture medium, including: FGF2 (25 or 50 ng/mL, Peprotech); BMP7 (200 ng/mL, Peprotech); Noggin (200 ng/mL, Peprotech); human WNT5A (100 ng/mL, R&D System); DKK1 (200 ng/mL, Peprotech); SU5402 (800 ng/mL, Calbiochem); and U0126 (20 nM; Calbiochem). The medium was changed every alternate day. At least 8 experiments for each treatment were performed. 

### 2.2. Morphological and Immunohistochemical Studies

For histological analysis, cultures were fixed in 4% paraformaldehyde (PFA), washed in PBS, dehydrated in acetone, and embedded in araldite. Semithin sections (1 µm) were stained with toluidine blue.

Chondrogenic differentiation was analyzed by Alcian blue staining (0.5% at pH 1.0) in cultures fixed with alcohol/acetic. 

Cell senescence was analyzed using the β-galactosidase activity assay [21] in cultures fixed overnight in 4% glutaraldehyde at pH 6. 

Immunolabeling was performed in samples grown on glass coverslip previously treated with 1 mg/mL fibronectin. At the desired stage, cultures were fixed for 3 h in 4% PFA, washed for 2 h in PBS-0.1% triton and blocked for 1.5 h in PBS-3% BSA and incubated overnight in the primary antibody. The samples were next washed for 30 min in PBS and incubated in the secondary antibody. We employed rabbit polyclonal anti-SOX9 (Merck-Millipore, AB5535) as a chondrogenic lineage marker and mouse monoclonal anti-γH2AX (Merck-Millipore, 05–636) to evaluate DNA damage. Actin labeling was performed with rhodamine-phalloidin (Sigma). 

Apoptotic cell death was analyzed by TUNEL assay using the in-situ cell death detection kit (Roche) following the manufacturer recommendations.

Histological observations were made with a LSM51O laser confocal microscope (Zeiss).

### 2.3. Flow Cytometry

Control and treated cultures were dissociated with trypsin EDTA (Lonza) and fixed in 90% ethanol. Samples containing one million cells were incubated overnight at 4 °C with 0.1% sodium citrate, 0.01% Triton X-100, and 0.1 mg/mL Propidium iodide (Sigma). The cell suspension was subjected to flow cytometry analysis in a Becton Dickinson FacsCanto cytometer and analyzed using Cell Quest software.

### 2.4. Quantification of Culture Growth

Culture growth was evaluated using the MTT assay. A total of 20 µL of 5 mg/mL tetrazolium salt was added to each culture well. After 2 h of further incubation the medium was replaced by dimethyl sulfoxide (DMSO) to lysate the tissue and the absorbance was evaluated by spectrophotometry at 570 nm. 

### 2.5. In situ Hybridization

The expression of growth factors tested in the study and a panel of limb mesodermal markers was confirmed by in situ hybridization in the cultured micromasses. Cultures fixed in 4% PFA were washed in PBT and treated with 7 µg/mL proteinase K for 2–5 min at 20 °C. Hybridization with digoxigenin-labeled anti-sense RNA probes was performed at 68 °C. Reactions were developed with BM-purple AP substrate (Roche). 

### 2.6. Epigenetic Analysis

We employed the Methylation-Sensitive Restriction Enzyme and Quantitative Polymerase Chain Reaction (MSRE-qPCR) technique to study changes in the methylation status of the *Sox9* and *Scleraxis* promoters. Genomic DNA samples from micromass cultures were extracted using NucleoSpin Tissue (Macherey–Nagel). In order to evaluate the methylation level of the target genes, we employed the EpiJET DNA Methylation Analysis Kit (MspI/HpaII) following manufacturer’s instructions. A total of 100 ng of purified genomic DNA were digested with the non-CpG-methylation-sensitive enzyme MspI or with the CpG-methylation-sensitive restriction enzyme HpaII for 6 h. Undigested Genomic DNA samples were treated as digested samples by replacing the volumes of the enzymes with DNase-free water. SYBRGreen-based qPCR was carried out in triplicates with a total volume of 20 μL per tube containing 2 μL of genomic DNA (MspI-digested, HpaII-digested, or undigested DNA), 0.4 μL of each specific primer, 10 μL of SYBR Select Master Mix (Life Technologies), and 7.2 μL of DNase-free water. Reactions were carried out in a StepOne Real Time System and analyzed by StepOne software v2.3 (Life Technologies). The relative percentage of methylated DNA was calculated according to the equation 2−ΔΔCt. Non-specific amplification was monitored by melting curve analysis of each reaction. Specific primers for CpG islands are indicated in Supplementary Table S1.

### 2.7. Real-Time Quantitative PCR (qPCR) for Gene Expression Analysis

Total RNA was extracted using the NucleoSpin RNA kit (Macherey–Nagel). First-strand cDNA was synthesized using random hexamers with the RevertAid RT Kit (Thermo Scientific). The cDNA concentration was adjusted to 0.5 μg/μL. SYBRGreen-based qPCR was performed employing SYBR Select Master Mix (Life technologies) using the CFX Connect Real-Time System (BioRad). Specificity was checked by the presence of single peaks in the dissociation curves. Gapdh was chosen as the normalizer. Mean values for gene expression fold changes were calculated relative to a calibrator according to the 2-ΔΔCt equation. qPCR chicken specific primers are indicated in Supplementary Table S1.

## 3. Results

Limb mesoderm micromass culture is an organoid-like tridimensional culture assay that closely recapitulates the chondrogenic differentiation of limb skeletal progenitors in vivo, including their spatial pattern and molecular regulatory signals [16,22]. To avoid employing a pool of cells with heterogeneous molecular signatures [15] and differences in their pattern of differentiation [18,23], in this study, we selected mesodermal progenitors obtained from the distal margin of stage 25 leg buds that express HoxA13 (Figure 1A–A´). All these progenitors have identical potential to form digits [24], but in normal development, they follow three distinct fates according to their position in the autopod: (i) forming digit cartilages at the digit ray position; (ii) forming the different autopodial fibrous connective tissues in the peridigital regions; or, (iii) undergoing programmed cell death in the interdigital spaces.

### 3.1. Differentiation, Proliferation, and Cell Death in Relation to the Presence or Absence of FBS in the Culture Medium

Our findings show that the micromass cultures obtained from limb bud mesoderm at stage 25 mimic, not only the chondrogenic differentiation, but also the degenerative events occurring in normal skeletal development. The difference is that cartilages in vitro take a nodular appearance rather than the radial arrangement of the digits observed in vivo, likely due to the polarized growth of the limb bud versus the uniform growth of the micromass culture. 

To select the conditions of our experiments we first evaluated differences in chondrogenesis, cell death, and cell proliferation in cultures growing in medium with or without FBS. In the first three days of culture, the growing tissue undergoes chondrogenic differentiation following a geometric nodular pattern that is believed to reflect the interactive molecular cues that govern normal skeletal development in vivo [22,25]. At Day 1 of culture in DMEM, the cells showed an irregular cell distribution, with zones of higher cell concentration and regions where cells were less compacted, but staining with Alcian blue, which is a cartilage matrix-specific dye, was negative at this stage (Figure 1B–B´). By Day 2 of culture, progenitors form regions of increased cell concentration that are intensely positive for SOX9 and show incipient nodules that are Alcian blue positive (Figure 1C–C´). By the third day of culture, the chondrogenic nodules expanded in number and size, forming numerous nodules positive for Alcian-blue staining (Figure 1D–D´) and SOX9 immunolabeling (Figure 1E). Cultures growing in a medium containing 10% FBS followed a similar sequence of chondrogenic differentiation, but the size of the cartilage nodules were significantly larger than those present in cultures lacking FBS (not shown). 

Analysis of cellular DNA contents by measuring propidium iodide uptake (Figure 1F) was performed in micromasses lacking FBS in the medium to avoid the interference of components present in the FBS with the treatments.

### 3.2. Patterned Cell Death and Cell Senescence

**Figure 2:**
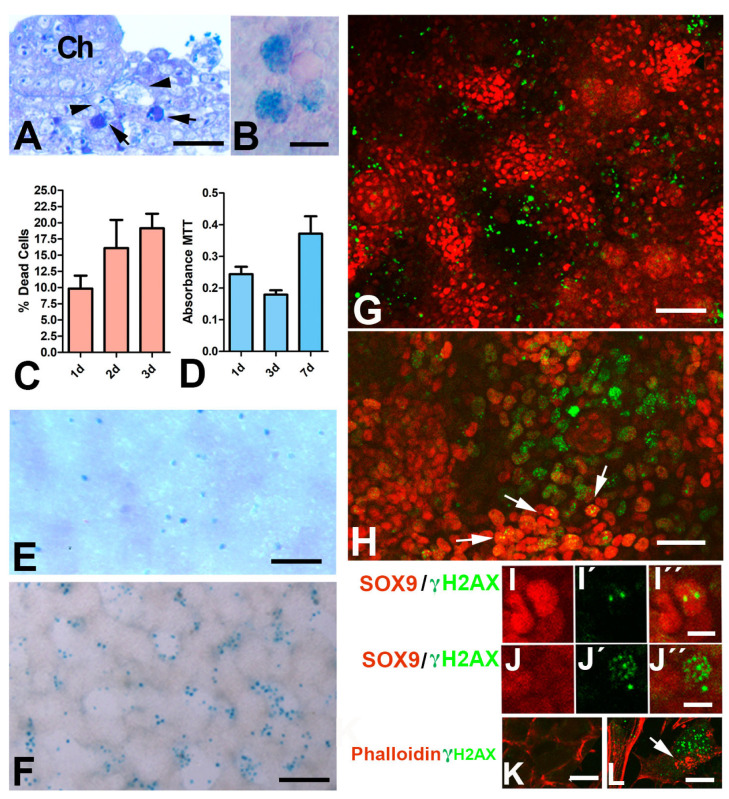
(**A**) Semithin section of a 3-day-old culture showing the presence of dark apoptotic cells (arrows) and vacuolated senescent cells (arrowheads) around a chrondrogenic nodule (Ch). (**B**) Detailed view of a micromass tissue showing senescent cells positive for SAβ-gal histochemistry (eosin counterstaining). (**C**) Graphic representation of the rate of cell death evaluated by flow cytometry during the first 3 days of culture grown in DMEM-only. (**D**) Total tissue mass of micromass cultures at Days 1, 3, and 7 evaluated by MTT staining. (**E**) Low-magnification view of 1-day culture after SAβ-gal histochemistry (dark blue staining) showing the distribution of senescent cells. (**F**) Perinodular restricted distribution of senescent cells positive for SAβ-gal in a 3-day-old micromass. (**G**) Optical section of 3-day-old micromass showing the arrangement of apoptosis (green TUNEL labeling) in the perinodular tissue. Note the intense positivity for SOX9 (red labeling) in chondrogenic aggregates. (**H**) A 3-day-old micromass section immunolabeled for γH2AX (green) and SOX9 (red) to show the preferential distribution of γH2AX in perinodular tissue. Note the very reduced nuclear yH2AX labeling in the aggregated cells highly positive for SOX9 (arrows). (**I**–**I”**) Detailed view of a SOX9-positive cell (**I**) with a couple of dots positive for γH2AX (**I’**). (**I”**) is the merged image. **(J**–**J”)** Detailed view of an internodular cell with poor SOX9 labeling (**J**) but massive γH2AX labeling (**J’**). (**J”**) is the merged image (**J”**). (**K**–**L**) Single channel and merged image of double labeling with γH2AX(green) and phalloidin (red) showing the aligned cytoplasmic actin filaments in healthy progenitors in contrast to the irregular aggregation of actin clumps (arrow) in the cytoplasm of cells highly positive for γH2AX (**K**). Scale bar in A and H = 20 µm; scale bar in B = 25 µm; scale bar in E and F = 300 µm; scale bar in G = 100 µm; scale bar I-J-K-L= 5 µm.

Tissue sections of three-day-old DMEM-only cultures revealed that degenerating cells were located around the differentiating cartilages (Figure 2A). These alterations included characteristic apoptotic cells, identifiable by the condensed nuclear morphology, and senescent cells [26] displaying a foamy and enlarged aspect because of vacuolization of the cytoplasm that was positive for senescence-associated β-galactosidase (SA-β-gal) labeling (Figure 2B). 

Analysis by flow cytometry revealed that the number of dead cells was similar in cultures of the same stage, i.e., the dead intensity was stage dependent (Figure 2C). On Day 1 of culture, 9.82% cells were dead. At Day 2, dead cells were 16.41% of the dissociated cells, and the rate of dead cells was 19.13% at Day 3. Consistent with these findings, the total tissue mass of the culture, evaluated by the MTT test, decreased during the first three days of culture (Figure 2D). In subsequent growth stages, the cartilage nodules expanded, showing a parallel increase in the total tissue mass of the culture as evaluated by MTT.

In the course of limb development in vivo, cell death and cell senescence remove the undifferentiated mesodermal progenitors of the autopod that separate the digit rays [20]. To assess whether the micromass cultures replicate the degenerative events occurring during skeletogenesis in vivo, the spatial pattern of cell degeneration was evaluated. SA-β-gal activity labeling was employed to detect cell senescence, and a TUNEL assay was used to detect apoptosis. At Day 1 of culture, senescent cells are scarce and predominate in the zones of lower cell density (Figure 2E). From Day 2 of culture, the distribution of degenerating cells replicated the degenerative process occurring in vivo. As shown in Figure 2F,G, both SA-β-gal-positive senescent cells (Figure 2F) and TUNEL-positive apoptotic cells (Figure 2G) showed a preferential arrangement in the internodular spaces. 

We previously observed that interdigital cell degeneration is preceded by intense DNA damage (DNAD) [27]. The histone variant H2AX is a key factor of the molecular cascade that cells activate by phosphorylation at serine 139 (γH2AX) to repair damaged DNA, and is a precise and precocious marker of the interdigital cells destined to die [28]. Consistent with these observations, we found a preferential distribution of cells with intense γH2AX immunolabeling in the undifferentiated perinodular tissue where apoptotic and senescent cells were distributed (Figure 2H,J). Reduced γH2AX labeling was also observed in some cells located in the core of the differentiating nodules of cartilage that were intensely positive for SOX9 (Figure 2I). This feature is consistent with the so-called noncanonical distribution of γH2AX observed during the differentiation of stem cells, which reflects chromatin epigenomic modifications associated with cell differentiation [29]. Additional markers of cell degeneration, such as a disorganized cytoskeleton [30], confirmed the internodular distribution of the dying cells (Figure 2K,L).

Overall, the above findings revealed a pattern of cell degeneration in the initial stages of differentiation of chondroprogenitors growing in micromass cultures that is reminiscent of the events associated with digit skeletogenesis in vivo.

### 3.3. FGFs Sensitize Chondroprogenitors to Dying Signals

During normal development, FGFs are considered survival signals for skeletal progenitors, but in vivo gain-of-function experiments via interdigital administration of an exogenous source of FGF showed transiently inhibited cell death [14,31,32]; but 24 h later, cell death increases dramatically [33]. Considering the double effect of FGFs inhibiting and promoting cell death, we performed different protocols of gain- and loss-of-function experiments of FGF signaling. 

**Figure 3:**
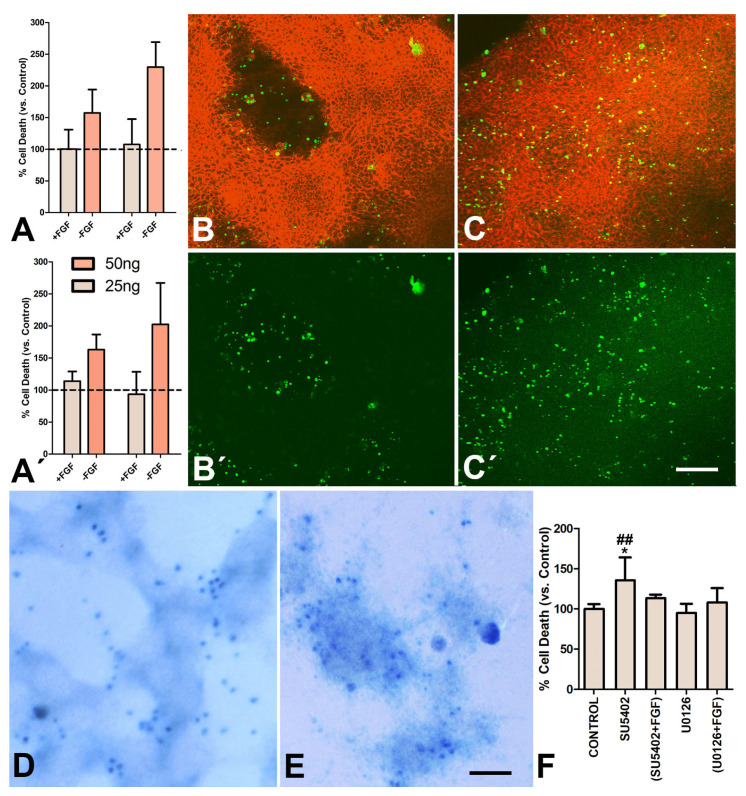
(**A**–**A’**) Graphic representation of the rate of cell death evaluated by flow cytometry in cultures treated for 2 days with 25 (light columns) or 50 ng/mL of FGF2 (red columns) to compare the effects of a continuous treatment with FGF (+FGF) and the effect of FGF withdrawal (-FGF) for 3 h (**A**) or 6 h (**A’**). The rate of cell death in the control untreated micromasses was considered 100 and is indicated by the dotted line. (**B**–**B’**) Confocal view of the perinodular arrangement of TUNEL-positive cells (green labeling) in a labeled control 2-days culture. The sample is also labeled with phalloidin (red labeling). (**B**) is a merged image, and (**B’**) shows only the green channel. (**C**–**C’**) Confocal view showing the widespread arrangement of TUNEL-positive cells (green labeling) after 3 h of FGF withdrawal in micromasses treated for 2 days with FGF2 (25 ngr/mL). Red labeling corresponds to phalloidin. (**C**) is a merged image, and (**C’**) shows only the green channel. (**D**,**E**) Detailed view of the SAβ-gal arrangement in control (**D**) and experimental micromasses subjected to FGF-withdrawal (**E**). Note the perinodular distribution of SAβ-gal in the control in contrast with the intranodular distribution in the experimental culture. (**F**) Graphic representation of the rate of cell death evaluated by flow cytometry in 2-day micromasses subjected to treatments with SU5402 (800 ng/mL), SU5402 plus 25 ng/mL of FGF2, U0126 (7.6 ng/mL), and U0126 plus FGF2. Scale bar in B−C´ = 250 µm; scale bar in D−E = 50 µm. * *p* < 0.05 (versus control); ##, *p* < 0.01 (versus SU5402+FGF2).

When FGF2 (25 or 50 ng/mL) was maintained for 48 h in the medium, the rate of cell death was similar to that of the control untreated cultures. However, removal of FGF from the medium for a period of 3 or 6 h at the end of the second day of culture increased cell death (Figure 3A–A’). Remarkably, in contrast to the perinodular distribution of apoptotic and senescent cells in the control cultures (Figure 3B,D), in FGF-treated cultures, degenerating cells were also associated with the regions of cell aggregation (Figure 3C,E).

To further characterize the role of FGF in cell degeneration in the cultured progenitors, we performed pharmacological inhibition of FGF signaling with SU5402 and U0126 during the first two days of culture (Figure 3F). Consistent with observations in the developing mouse limb [32], cultures growing in a medium containing 800 ng/mL SU5402, which is a selective inhibitor of FGF receptor tyrosine kinase activity, increased cell death by 43%. Consistent with the anti-FGF influence of SU5402, the intensity of cell death by SU5402 was attenuated in the presence of exogenous FGF2 in the medium (Figure 3F). 

In contrast to SU5402, the addition of the MAPK inhibitor U0126 at a dose of 7.6 ng/mL (20 nM), alone or in combination with FGF, did not modify the rate or the intensity of cell death (Figure 3F). Consistent with studies in vivo [32], U0126 at a much higher concentration (50µM vs. 20 nM) increased cell death in the cultures (not shown). 

### 3.4. Epigenetic Influence of FGFs

**Figure 4:**
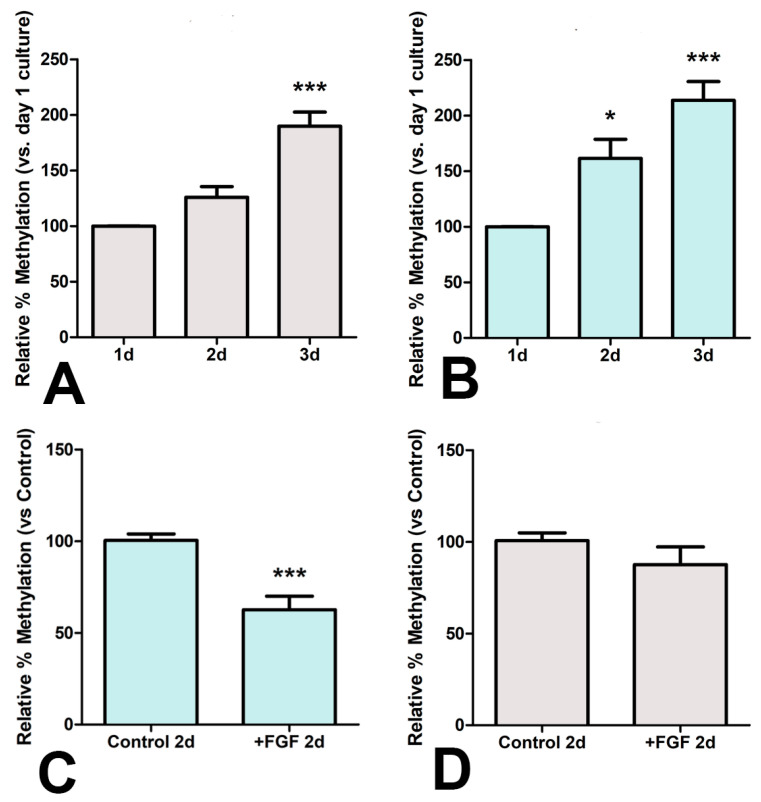
(**A**,**B**) Changes in the rate of cytosine methylation of the *Scleraxis* (**A**) and *Sox9* (**B**) promoters during the first three days of micromass culture. (**C**,**D**) Changes in the rate of cytosine methylation of the *Sox9* (**C**) and *Scleraxis* (**D**) promoters during the first two days of culture induced by the addition of FGF2 (25 ng/mL) to the culture medium. * *p* < 0.05; *** *p* < 0.001.

We have previously observed an intense positive influence of FGF signaling on the limb expression of the epigenetic modulators *Uhrf1* and *Uhrf2*. These genes encode factors that modulate transcriptional regulation and death sensitivity via chromatin modifications [34]. In addition, FGFs transiently delay chondrogenic differentiation of limb skeletal progenitors as well as counteract the permanent antichondrogenic influence of WNT signaling secondary to the cytosine methylation of the *Sox9* promoter [11]. Hence, to explore the importance of epigenetic modifications of differentiating progenitors in the outcome of the micromass cultures, we analyzed by MSRE-qPCR the cytosine methylations (5-mC) in the promoter of *Sox9* and *Scleraxis* (*Sclx*) genes as master genes for chondrogenic and fibrogenic differentiation of the skeletal progenitors, respectively. In the control cultures, the promoter of *Sclx* maintained a low percentage of 5-mCs (0.24% at Day 1), and the rate increased moderately during the three days analyzed in our study (Figure 4A). In contrast to *Sclx*, the promoter of *Sox9* showed higher levels of methylation (3.97% of 5-mC at Day 1) that also increased in the course of culture (Figure 4B). The addition of FGF2 (25 ng/mL) to the medium significantly decreased the methylation of the *Sox9* promoter (Figure 4C) but not that of *Scleraxis* (Figure 4D).

### 3.5. Transcriptional Effects of FGFs

The survival role of FGFs deduced from SU5402 treatments, along with the intense degeneration caused by the removal of FGFs when the cultures were grown in a medium containing exogenous FGFs, prompted us to characterize the transcriptional modifications induced by FGFs in the skeletal progenitors by qPCR. For this purpose we selected a panel of genes associated with: chondrogenic (*Sox9*) and fibrogenic (*Scleraxis*) differentiation; mesodermal cell undifferentiation (*Msx1, Msx2, Oct4*); cell death (*Bcl2, Bak1*); cell senescence (*p21, GBL1, Mmp2, Cathepsin D, Il-6*); epigenetic regulators active in the embryonic limb (*Dnmt1; Dnmt3A; Dnmt3B; TET3; HDAC2; HDAC3; HDAC8; and Prmt5*); and components of the major signaling pathways active during digit skeletogenesis (*Bmp2, Bmp4, Bmp5, Bmp7, Noggin, FgfR1, FgfR2, FgfR3, FgfR4, Wnt5a, and Dkk1*). The efficiency of FGF treatments was confirmed by detecting the regulation of *Sprouty1* [35]. 

As shown in Table 1, no major changes were observed in genes associated with differentiation or undifferentiation in the FGF-treated cultures. Changes were not appreciated in *Wnt5a*. Minor changes were observed in FGF receptors and in the selected epigenetic regulators, in addition to significant down-regulation of *FgfR2, Dnmt1*, and *HDAC3*. As described in other experimental settings [36], various BMP ligands involved in programmed cell death [37], including *Bmp2, Bmp4 and Bmp7*, were intensely upregulated, while *Bmp5* and the BMP antagonist *Noggin* were down-regulated. *Dkk1* was also up-regulated.

Remarkably, the most important changes were detected in the expression of apoptotic and cell senescence factors, including members of the senescence-associated secretome (SASP). Thus, the pro-apoptotic factor *Bak1* and *Cyclin-Dependent Kinase Inhibitor 1* (*p21*), which is considered the main regulator of developmental senescence, and *Cathepsin D* and *Il-6* were upregulated by more than twofold, while *Bcl2*, which is an antiapoptotic factor, was not regulated at significant levels. Beta-galactosidase (*GBL1*) was up-regulated but without reaching statistically significant levels.

### 3.6. FGF2 Modifies the Response of Cultured Progenitors to BMP- and WNT- Signaling

**Figure 5:**
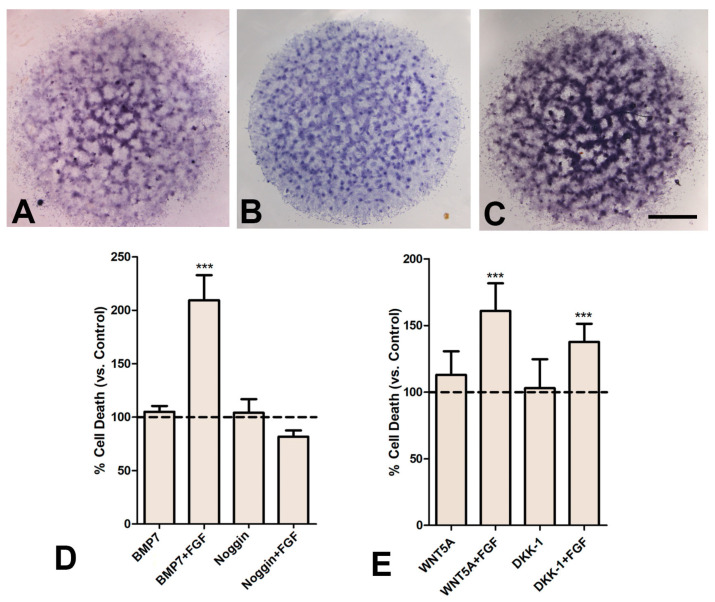
(**A**–**C**) In situ hybridizations of 2-day-old micromass cultures showing the expression of *Bmp7* (**A**), *Wnt5a* (**B**), and *Dkk1* (**C**). (**D**) Graphic representation of the rate of cell death evaluated by flow cytometry in cultures treated for 2 days with BMP7 (200 ng/mL); BMP7 plus FGF2 (25 ng/mL); NOGGIN (200 ng/mL); and NOGGIN plus FGF2 (25 ng/mL). The rate of cell death in untreated control cultures was considered to be 100% and indicated by the dotted line. (**E**) Graphic representation of the rate of cell death evaluated by flow cytometry in cultures treated for 2 days with WNT5A (100 ng/mL), WNT5A plus FGF2(25 ng/mL), DKK1 (200 ng/mL), and DKK-1 plus FGF2 (25 ng/mL). The rate of cell death in untreated control cultures was considered to be 100 and is indicated by the dotted line. Scale bar in A, B, C = 0.5 mm. *** *p* < 0.001.

To analyze the crosstalk between FGF signaling and other signals active in the developing limb, we examined the effects of combined treatments with FGF2 and growth factors that regulate chondrogenic differentiation and cell death in the developing limb [38]. We selected *Bmp7, Wnt5a*, and the WNT antagonist *Dkk1* because they are highly expressed in the micromass cultures (Figure 5A–C) and show overlapping expression with regions of programmed cell death in vivo.

#### 3.6.1. The FGF/BMP Axis

BMPs have been identified as the apoptotic triggering signals for the undifferentiated embryonic limb mesoderm [37,39] but they are also growth-promoting signals for prechondrogenic aggregates [12,37,38,40,41]. Our findings revealed that the proapoptotic influence of BMPs in two-day cultures was only detected in BMP7 treatments combined with FGF2 (182% double-treated vs. 108% BMP7-treated-only: Figure 5D). In a complementary fashion, the apoptotic-protective effect of the BMP antagonist NOGGIN also required combined treatments with FGFs (Figure 5D). These findings suggest a permissive influence of FGF signaling on the regulation of apoptosis driven by BMP signaling.

#### 3.6.2. The FGF/WNT Axis

WNT signaling exerts a critical role in limb outgrowth and skeletogenesis [38,42,43]. Members of the family are believed to exert a protective role against programmed cell death associated with the crosstalk between FGF and BMP signaling rather than a direct influence in the degenerative process [38]. However, WNT5a, which signals via noncanonical pathway [44], and the WNT antagonist DKK1, which inhibits Wnt/β-catenin signaling, have been associated with cell death because they show specific expression domains in the areas of interdigital cell death [45].

In the micromass assay, endogenous expression of *Wnt5a* and *Dkk1* was confirmed by in situ hybridization (Figure 5 B,C) prior to analysis of the influence of their exogenous administration to the medium. As observed in vivo [46], individual addition of WNT5A (100 ng/mL) or DKK1 (200 ng/mL) to the culture medium did not significantly increase the intensity of cell death. However, both treatments increased cell death when they were applied in combination with FGF2 (Figure 5E). 

## 4. Discussion

The culture of limb skeletal progenitors at high concentrations recapitulates embryonic skeletogenesis and is controlled in a fashion similar to that in vivo [16,22,47]. Our findings reveal that the differentiation of progenitors in this assay is accompanied by a patterned process of cell degeneration that delimits the initial regions of prechondrogenic aggregation. Cell death modulates tissue differentiation and morphogenesis during embryonic development of most organs, including limb skeletogenesis [4]. Our present results reveal that a comparable degeneration process accompanies the differentiation of chondroprogenitors in vitro. The initial formation of cartilaginous nodules in the micromass culture assay is accompanied by the elimination of progenitors located in the contour of the differentiating cartilage nodules. Remarkably, degeneration in this assay exhibits the same mechanistic features detected during in vivo tissue remodeling [20,27], which includes initial DNA damage, followed by the appearance of TUNEL-positive apoptosis and cell senescence positive for beta-galactosidase at pH 6. Apoptosis mediated by caspases is the major dying mechanism responsible for programmed cell death in embryonic systems. However, during the last decade cell senescence has gained interest in embryonic studies [48,49,50,51,52] because it may represent a nonapoptotic cell degeneration involving the active participation of lysosomes [20]. The combined participation of apoptosis and cell senescence in this in vitro assay supports a similar relevance of both mechanisms for tissue remodeling in the course of tissue differentiation. 

The internodular pattern of distribution of cell death and cell senescence in the control cultures is consistent with observations in monolayer cultures [18], suggesting that prechondrogenic aggregation serves a survival function for chondroprogenitors. A tempting explanation for the patterned dying process in the micromass culture assay is that the intense cell rearrangement involved in the formation of chondrogenic nodules might generate mechanical cues that contribute to modulating degeneration [53]. It has been shown that mechanical stress generated during the initial stages of cell aggregation [54,55] together with BMP signaling [56,57] regulate the expression of *Sox9* [58], a master chondrogenic factor that protects chondroprogenitors from cell death [59]. 

Experiments in vivo were suggestive of an antagonist effect of FGF and BMP signaling [12]. Our findings revealed a survival influence of FGF signaling on the differentiating progenitors accompanied by the sensitization of cells to other apoptotic stimuli. Thus, cell death increased dramatically when FGF signaling was blocked by treatments with the FGF inhibitor SU5402 and also by addition and subsequent removal of FGF2 from the culture medium. Remarkably, the difference in the internodular distribution of senescent and apoptotic cells in control cultures indicates that increased apoptosis and senescence due to FGF removal take place at the expense of the peripheral cells of the prechondrogenic aggregates, which survive and form cartilage in the control untreated cultures. These observations support the role of FGF signaling in maintaining chondroprogenitor survival and proliferation, favoring either subsequent differentiation [10,11] or removal by cell death [33], depending on complementary signals active in specific spatial and temporal patterns. FGFs are master factors that maintain stemness in mesenchymal stem cells [60] and are responsible for controlling embryonic limb outgrowth [61,62,63]. Furthermore, in the developing limb there is a temporal association between the extinction of *Fgf8* expression in the AER and establishment of the areas of interdigital cell death [32,64]. 

The transcriptional changes induced by FGF treatments in the micromass culture assay included the upregulation of factors that participate in the physiological degeneration of the interdigital tissue in the embryonic limb. The increased presence of these factors in the progenitors when the survival influence of FGFs is extinguished may contribute to the onset of degeneration. Among such degeneration-promoting factors upregulated by FGF-treatments are: *p21*, a specific marker of cell senescence [49], and *Bak1*, which is responsible for the activation of the mitochondrial apoptotic pathway [32]. The influence of these factors may be counteracted by protective factors regulated by FGF while present in the culture medium. An additional mechanism underlying the effects of FGF signaling is the changes in the epigenetic profile of progenitors. It has been shown that FGFs protect progenitors from irreversible methylation of the promoter of *Sox9*, favoring their subsequent differentiation into cartilage upon contact with pro-chondrogenic signals [11]. Our observations reveal a negative influence of FGF signaling on the expression of *DNA methyltransferase 1 (Dnmt1)* and *histone deacetylase 3 (HDAC3)*, accompanied by a decrease in the methylation of the *Sox9* promoter. *DNA methyl transferase 3A (Dnmt3A)* was also downregulated but did not show statistical significance. These changes would result in chromatin architectural modifications likely making progenitors more susceptible to DNA damage. This hypothesis is consistent with the importance of SOX9 for the survival of chondrogenic [59,65] and other cell populations [66]. 

A major finding observed in the present study is the differential response of growing progenitors to BMP and WNT signaling depending on the presence in the medium of FGF2. There is compelling evidence supporting a central role of BMPs as death triggering factors for embryonic limb programmed cell death, including regression of the AER that is the source of FGFs [12,27,37,38,39,40,41]. However, similar experimental approaches demonstrate an opposite role of BMP signaling in promoting the formation and growth of chondrogenic aggregates [12,37,57]. These contradictory functions of BMPs generated controversy about the physiological hierarchy of the death-triggering machinery during interdigit regression [67]. Under the experimental conditions of our study, BMP7 exerted a mild pro-apoptotic influence in the cultures that was potentiated when exogenous FGF2 was added to the culture medium. Considering that suppression of FGF signaling induces cell death and cell senescence in the cultures, our findings indicate that FGF cessation and BMP activation are two complementary pathways accounting for cell death of progenitors.

Wnt/β-catenin canonical signaling inhibits chondrogenesis via epigenetic silencing of the promoter of *Sox9* [11] and stimulates limb outgrowth by promoting the expression of FGFs in the AER, which in turn maintain progenitor proliferation and survival [68]. The protective role of WNT signaling appears to be abolished in the areas of cell death due to the expression of the WNT antagonist *Dkk1* which is expressed under the control of BMPs [45,68,69]. However, in an opposite fashion, cell death in the developing hindbrain is inhibited by WNT antagonists via upregulation of BMP signaling [70]. Our findings confirm the interaction between FGF and WNT signaling in the control of cell death [45]. We show that neither WNT5A nor DKK treatments modified cell death at significant levels in the absence of exogenous FGFs, but both factors induced apoptosis and cell senescence when they were combined with FGF2. It must be emphasized that WNT5A is a member of the WNT family, which signals via noncanonical inhibiting Wnt/β-catenin canonical signaling in the developing limb [44]. Together, these results emphasize the importance of tuning WNT signaling to control the balance between differentiation and cell death proposed by Kumar and Lassar [11].

## Figures and Tables

**Table 1:** Gene expression fold change values in FGF-treated versus control untreated, two-day-old micromass cultures. Fold change values are expressed as Mean ± SD. * *p* < 0.05; ** *p* < 0.01; *** *p* < 0.001. ^a^ very low expression level. Bold is regulated genes.

Gene.	FGF2 vs Control.	Gene.	FGF2 vs Control.
Differentiation markers.		FGF signaling.	
*Sclx*	1.06 ± 0.48	***Sprouty1***	21.46 ± 13.53 ***
*Sox9*	1.40 ± 0.99	*FgfR1*	1.11 ± 0.46
Senescence markers.		***FgfR2***	0.54 ± 0.20 ***
***p21***	2.70 ± 1.08 ***	*FgfR3*	0.78 ± 0.50
*GBL1*	1.42 ± 0.46	*FgfR4*	0.94 ± 0.47
***Cathepsin D***	2.38 ± 0.84 *	BMP signaling.	
*Mmp2*	1.30 ± 0.26	***Bmp2***	2.30 ± 0.86 **
***Il-6***	2.59 ± 0.93 *^,a^	***Bmp4***	2.67 ± 1.05 **
Cell Death markers.		***Bmp5***	0.40 ± 0.23 ***
*Bcl2*	1.19 ± 0.46	***Bmp7***	3.75 ± 1.63 ***
***Bak1***	2.56 ± 0.91 ***	*Noggin*	0.73 ± 0.52
Undifferentiation markers.		WNT signaling.	
*Msx1*	1.27 ± 0.58	*Wnt5a*	0.90 ± 0.34
*Msx2*	1.34 ± 0.57	***Dkk1***	4.71 ± 1.80 **
*Oct4*	1.40 ± 0.23 ^a^		
Epigenetic modulators.			
***Dnmt1***	0.56 ± 0.24 *		
*Dnmt3A*	0.61 ± 0.31		
*Dnmt3B*	0.96 ± 0.35		
*TET3*	0.69 ± 0.19		
*HDAC2*	0.68 ± 0.27	Note: ^a^ very low expression level CTs > 30 vs Gapdh (CT ≈ 18).	
***HDAC3***	0.55 ± 0.24 *		
*HDAC8*	0.77 ± 0.26		
*Prmt5*	1.08 ± 0.10		

## Data Availability

The data presented in this study are available on request from the corresponding author.

## Supplementary Materials

The following supporting information can be downloaded at: https://www.mdpi.com/article/10.3390/cells12010175/s1, Supplementary Table S1: qPCR and MSRE-qPCR chicken specific primers.

## References

[1] Glücksmann A. (1951). Cell Deaths in Normal Vertebrate Ontogeny. Biol. Rev..

[2] Soteriou D., Fuchs Y. (2018). A Matter of Life and Death: Stem Cell Survival in Tissue Regeneration and Tumour Formation. Nat. Rev. Cancer.

[3] Nie X., Luukko K., Fjeld K., Kvinnsland I.H., Kettunen P. (2005). Developmental Expression of Dkk1-3 and Mmp9 and Apoptosis in Cranial Base of Mice. J. Mol. Histol..

[4] Montero J.A., Lorda-Diez C.I., Sanchez-Fernandez C., Hurle J.M. (2020). Cell Death in the Developing Vertebrate Limb: A Locally Regulated Mechanism Contributing to Musculoskeletal Tissue Morphogenesis and Differentiation. Dev. Dyn..

[5] DeLise A.M., Fischer L., Tuan R.S. (2000). Cellular Interactions and Signaling in Cartilage Development. Osteoarthr. Cartil..

[6] Yoon B.S., Ovchinnikov D.A., Yoshii I., Mishina Y., Behringer R.R., Lyons K.M. (2005). Bmpr1a and Bmpr1b Have Overlapping Functions and Are Essential for Chondrogenesis in Vivo. Proc. Natl. Acad. Sci. USA.

[7] Mina M., Gluhak J., Upholt W.B., Kollar E.J., Rogers B. (1995). Experimental Analysis of Msx-1 and Msx-2 Gene Expression during Chick Mandibular Morphogenesis. Dev. Dyn. Off. Publ. Am. Assoc. Anat..

[8] Shim M., Foley J., Anna C., Mishina Y., Eling T. (2010). Embryonic Expression of Cyclooxygenase-2 Causes Malformations in Axial Skeleton. J. Biol. Chem..

[9] Moftah M.Z., Downie S.A., Bronstein N.B., Mezentseva N., Pu J., Maher P.A., Newman S.A. (2002). Ectodermal FGFs Induce Perinodular Inhibition of Limb Chondrogenesis in Vitro and in Vivo via FGF Receptor 2. Dev. Biol..

[10] Kumar M., Ray P., Chapman S.C. (2012). Fibroblast Growth Factor and Bone Morphogenetic Protein Signaling Are Required for Specifying Prechondrogenic Identity in Neural Crest-Derived Mesenchyme and Initiating the Chondrogenic Program. Dev. Dyn..

[11] Kumar D., Lassar A.B. (2014). Fibroblast Growth Factor Maintains Chondrogenic Potential of Limb Bud Mesenchymal Cells by Modulating DNMT3A Recruitment. Cell Rep..

[12] Buckland R.A., Collinson J.M., Graham E., Davidson D.R., Hill R.E. (1998). Antagonistic Effects of FGF4 on BMP Induction of Apoptosis and Chondrogenesis in the Chick Limb Bud. Mech. Dev..

[13] Semba I., Nonaka K., Takahashi I., Takahashi K., Dashner R., Shum L., Nuckolls G.H., Slavkin H.C. (2000). Positionally-Dependent Chondrogenesis Induced by BMP4 Is Co-Regulated by Sox9 and Msx2. Dev. Dyn..

[14] Zhao W., Allen S., Dhoot G.K. (2007). FGF Mediated Sulf1 Regulation. FEBS Lett..

[15] Reinhardt R., Gullotta F., Nusspaumer G., Ünal E., Ivanek R., Zuniga A., Zeller R. (2019). Molecular Signatures Identify Immature Mesenchymal Progenitors in Early Mouse Limb Buds That Respond Differentially to Morphogen Signaling. Development.

[16] Klumpers D.D., Mooney D.J., Smit T.H. (2015). From Skeletal Development to Tissue Engineering: Lessons from the Micromass Assay. Tissue Eng. Part B. Rev..

[17] Raspopovic J., Marcon L., Russo L., Sharpe J. (2014). Digit Patterning Is Controlled by a Bmp-Sox9-Wnt Turing Network Modulated by Morphogen Gradients. Science.

[18] Omi M., Sato-Maeda M., Ide H. (2000). Role of Chondrogenic Tissue in Programmed Cell Death and BMP Expression in Chick Limb Buds. Int. J. Dev. Biol..

[19] Yamashita A., Krawetz R., Rancourt D.E. (2009). Loss of Discordant Cells during Micro-Mass Differentiation of Embryonic Stem Cells into the Chondrocyte Lineage. Cell Death Differ..

[20] Montero J.A., Lorda-Diez C.I., Hurle J.M. (2020). Confluence of Cellular Degradation Pathways During Interdigital Tissue Remodeling in Embryonic Tetrapods. Front. Cell Dev. Biol..

[21] Debacq-Chainiaux F., Erusalimsky J.D., Campisi J., Toussaint O. (2009). Protocols to Detect Senescence-Associated Beta-Galactosidase (SA-Βgal) Activity, a Biomarker of Senescent Cells in Culture and in Vivo. Nat. Protoc..

[22] Rolfe R.A., Shea C.A., Murphy P. (2022). Geometric Analysis of Chondrogenic Self-Organisation of Embryonic Limb Bud Cells in Micromass Culture. Cell Tissue Res..

[23] Downie S.A., Newman S.A. (1994). Morphogenetic Differences between Fore and Hind Limb Precartilage Mesenchyme: Relation to Mechanisms of Skeletal Pattern Formation. Dev. Biol..

[24] Merino R., Macias D., Gañan Y., Rodriguez-Leon J., Economides A.N., Rodriguez-Esteban C., Izpisua-Belmonte J.C., Hurle J.M. (1999). Control of Digit Formation by Activin Signalling. Development.

[25] Kiskowski M.A., Alber M.S., Thomas G.L., Glazier J.A., Bronstein N.B., Pu J., Newman S.A. (2004). Interplay between Activator–Inhibitor Coupling and Cell-Matrix Adhesion in a Cellular Automaton Model for Chondrogenic Patterning. Dev. Biol..

[26] Yadav P., Chatterjee K., Saini D.K. (2021). Senescent Cells in 3D Culture Show Suppressed Senescence Signatures. Biomater. Sci..

[27] Montero J.A., Sanchez-Fernandez C., Lorda-Diez C.I., Garcia-Porrero J.A., Hurle J.M. (2016). DNA Damage Precedes Apoptosis during the Regression of the Interdigital Tissue in Vertebrate Embryos. Sci. Rep..

[28] Sanchez-Fernandez C., Lorda-Diez C.I., Hurlé J.M., Montero J.A. (2020). The Methylation Status of the Embryonic Limb Skeletal Progenitors Determines Their Cell Fate in Chicken. Commun. Biol..

[29] Orlando L., Tanasijevic B., Nakanishi M., Reid J.C., García-Rodríguez J.L., Chauhan K.D., Porras D.P., Aslostovar L., Lu J.D., Shapovalova Z. (2021). Phosphorylation State of the Histone Variant H2A.X Controls Human Stem and Progenitor Cell Fate Decisions. Cell Rep..

[30] Zuzarte-Luis V., Berciano M.T., Lafarga M., Hurlé J.M. (2006). Caspase Redundancy and Release of Mitochondrial Apoptotic Factors Characterize Interdigital Apoptosis. Apoptosis.

[31] Macias D., Gañan Y., Ros M.A., Hurle J.M. (1996). In Vivo Inhibition of Programmed Cell Death by Local Administration of FGF-2 and FGF-4 in the Interdigital Areas of the Embryonic Chick Leg Bud. Anat. Embryol..

[32] Hernández-Martínez R., Castro-Obregón S., Covarrubias L. (2009). Progressive Interdigital Cell Death: Regulation by the Antagonistic Interaction between Fibroblast Growth Factor 8 and Retinoic Acid. Development.

[33] Montero J.A., Gañan Y., Macias D., Rodriguez-Leon J., Sanz-Ezquerro J.J., Merino R., Chimal-Monroy J., Nieto M.A., Hurle J.M. (2001). Role of FGFs in the Control of Programmed Cell Death during Limb Development. Development.

[34] Sanchez-Fernandez C., Lorda-Diez C.I., García-Porrero J.A., Montero J.A., Hurlé J.M. (2019). UHRF Genes Regulate Programmed Interdigital Tissue Regression and Chondrogenesis in the Embryonic Limb. Cell Death Dis..

[35] Ornitz D.M., Itoh N. (2015). The Fibroblast Growth Factor Signaling Pathway. Wiley Interdiscip. Rev. Dev. Biol..

[36] Yang W., Cao Y., Zhang Z., Du F., Shi Y., Li X., Zhang Q. (2018). Targeted Delivery of FGF2 to Subchondral Bone Enhanced the Repair of Articular Cartilage Defect. Acta Biomater..

[37] Macias D., Gañan Y., Sampath T.K., Piedra M.E., Ros M.A., Hurle J.M. (1997). Role of BMP-2 and OP-1 (BMP-7) in Programmed Cell Death and Skeletogenesis during Chick Limb Development. Development.

[38] Díaz-Hernández M.E., Galván-Hernández C.I., Marín-Llera J.C., Camargo-Sosa K., Bustamante M., Wischin S., Chimal-Monroy J. (2021). Activation of the WNT-BMP-FGF Regulatory Network Induces the Onset of Cell Death in Anterior Mesodermal Cells to Establish the ANZ. Front. Cell Dev. Biol..

[39] Kaltcheva M.M., Anderson M.J., Harfe B.D., Lewandoski M. (2016). BMPs Are Direct Triggers of Interdigital Programmed Cell Death. Dev. Biol..

[40] Bandyopadhyay A., Tsuji K., Cox K., Harfe B.D., Rosen V., Tabin C.J. (2006). Genetic Analysis of the Roles of BMP2, BMP4, and BMP7 in Limb Patterning and Skeletogenesis. PLoS Genet..

[41] Zuzarte-Luís V., Montero J.A., Rodriguez-León J., Merino R., Rodríguez-Rey J.C., Hurlé J.M. (2004). A New Role for BMP5 during Limb Development Acting through the Synergic Activation of Smad and MAPK Pathways. Dev. Biol..

[42] Villacorte M., Suzuki K., Hayashi K., de Sousa Lopes S.C., Haraguchi R., Taketo M.M., Nakagata N., Yamada G. (2010). Antagonistic Crosstalk of Wnt/Beta-Catenin/Bmp Signaling within the Apical Ectodermal Ridge (AER) Regulates Interdigit Formation. Biochem. Biophys. Res. Commun..

[43] Gao B., Ajima R., Yang W., Li C., Song H., Anderson M.J., Liu R.R., Lewandoski M.B., Yamaguchi T.P., Yang Y. (2018). Coordinated Directional Outgrowth and Pattern Formation by Integration of Wnt5a and Fgf Signaling in Planar Cell Polarity. Dev..

[44] Topol L., Jiang X., Choi H., Garrett-Beal L., Carolan P.J., Yang Y. (2003). Wnt-5a Inhibits the Canonical Wnt Pathway by Promoting GSK-3-Independent Beta-Catenin Degradation. J. Cell Biol..

[45] Grotewold L., Rüther U. (2002). The Wnt Antagonist Dickkopf-1 Is Regulated by Bmp Signaling and c-Jun and Modulates Programmed Cell Death. EMBO J..

[46] Farrera-Hernández A., Marín-Llera J.C., Chimal-Monroy J. (2021). WNT5A-Ca2+-CaN-NFAT Signalling Plays a Permissive Role during Cartilage Differentiation in Embryonic Chick Digit Development. Dev. Biol..

[47] Christley S., Alber M.S., Newman S.A. (2007). Patterns of Mesenchymal Condensation in a Multiscale, Discrete Stochastic Model. PLOS Comput. Biol..

[48] de Mera-Rodríguez J.A., Álvarez-Hernán G., Gañán Y., Martín-Partido G., Rodríguez-León J., Francisco-Morcillo J. (2021). Is Senescence-Associated β-Galactosidase a Reliable in Vivo Marker of Cellular Senescence During Embryonic Development?. Front. Cell Dev. Biol..

[49] Lorda-Diez C.I., Garcia-Riart B., Montero J.A., Rodriguez-León J., Garcia-Porrero J.A., Hurlé J.M. (2015). Apoptosis during Embryonic Tissue Remodeling Is Accompanied by Cell Senescence. Aging.

[50] Muñoz-Espín D., Cañamero M., Maraver A., Gómez-López G., Contreras J., Murillo-Cuesta S., Rodríguez-Baeza A., Varela-Nieto I., Ruberte J., Collado M. (2013). XProgrammed Cell Senescence during Mammalian Embryonic Development. Cell.

[51] Rhinn M., Ritschka B., Keyes W.M. (2019). Cellular Senescence in Development, Regeneration and Disease. Development.

[52] Varela-Nieto I., Palmero I., Magariños M. (2019). Complementary and Distinct Roles of Autophagy, Apoptosis and Senescence during Early Inner Ear Development. Hear. Res..

[53] De Belly H., Paluch E.K., Chalut K.J. (2022). Interplay between Mechanics and Signalling in Regulating Cell Fate. Nat. Rev. Mol. Cell Biol..

[54] Parada C., Banavar S.P., Khalilian P., Rigaud S., Michaut A., Liu Y., Joshy D.M., Campàs O., Gros J. (2022). Mechanical Feedback Defines Organizing Centers to Drive Digit Emergence. Dev. Cell.

[55] Weng S., Huebner R.J., Wallingford J.B. (2022). Convergent Extension Requires Adhesion-Dependent Biomechanical Integration of Cell Crawling and Junction Contraction. Cell Rep..

[56] Barna M., Niswander L. (2007). Visualization of Cartilage Formation: Insight into Cellular Properties of Skeletal Progenitors and Chondrodysplasia Syndromes. Dev. Cell.

[57] Lim J., Tu X., Choi K., Akiyama H., Mishina Y., Long F. (2015). BMP-Smad4 Signaling Is Required for Precartilaginous Mesenchymal Condensation Independent of Sox9 in the Mouse. Dev. Biol..

[58] Juhász T., Matta C., Somogyi C., Katona É., Takács R., Soha R.F., Szabó I.A., Cserháti C., Sződy R., Karácsonyi Z. (2014). Mechanical Loading Stimulates Chondrogenesis via the PKA/CREB-Sox9 and PP2A Pathways in Chicken Micromass Cultures. Cell. Signal..

[59] Akiyama H., Chaboissier M.C., Martin J.F., Schedl A., De Crombrugghe B. (2002). The Transcription Factor Sox9 Has Essential Roles in Successive Steps of the Chondrocyte Differentiation Pathway and Is Required for Expression of Sox5 and Sox6. Genes Dev..

[60] Coutu D.L., Galipeau J. (2011). Roles of FGF Signaling in Stem Cell Self-Renewal, Senescence and Aging. Aging.

[61] Martin G.R. (1998). The Roles of FGFs in the Early Development of Vertebrate Limbs. Genes Dev..

[62] Verheyden J.M., Lewandoski M., Deng C., Harfe B.D., Sun X. (2005). Conditional Inactivation of Fgfr1 in Mouse Defines Its Role in Limb Bud Establishment, Outgrowth and Digit Patterning. Development.

[63] Xu X., Weinstein M., Li C., Naski M., Cohen R.I., Ornitz D.M., Leder P., Deng C. (1998). Fibroblast Growth Factor Receptor 2 (FGFR2)-Mediated Reciprocal Regulation Loop between FGF8 and FGF10 Is Essential for Limb Induction. Development.

[64] Gañan Y., Macias D., Basco R.D., Merino R., Hurle J.M. (1998). Morphological Diversity of the Avian Foot Is Related with the Pattern of Msx Gene Expression in the Developing Autopod. Dev. Biol..

[65] Chimal-Monroy J., Rodriguez-Leon J., Montero J.A., Gañan Y., Macias D., Merino R., Hurle J.M. (2003). Analysis of the Molecular Cascade Responsible for Mesodermal Limb Chondrogenesis: Sox Genes and BMP Signaling. Dev. Biol..

[66] Ma Y., Shepherd J., Zhao D., Bollu L.R., Tahaney W.M., Hill J., Zhang Y., Mazumdar A., Brown P.H. (2020). SOX9 Is Essential for Triple-Negative Breast Cancer Cell Survival and Metastasis. Mol. Cancer Res..

[67] Hernández-Martínez R., Covarrubias L. (2011). Interdigital Cell Death Function and Regulation: New Insights on an Old Programmed Cell Death Model. Dev. Growth Differ..

[68] Chimal-Monroy J., Montero J.A., Gañan Y., Macias D., Garcia-Porrero J.A., Hurle J.M. (2002). Comparative Analysis of the Expression and Regulation of Wnt5a, Fz4, and Frzb1 during Digit Formation and in Micromass Cultures. Dev. Dyn..

[69] Mukhopadhyay M., Shtrom S., Rodriguez-Esteban C., Chen L., Tsukui T., Gomer L., Dorward D.W., Glinka A., Grinberg A., Huang S.P. (2001). Dickkopf1 Is Required for Embryonic Head Induction and Limb Morphogenesis in the Mouse. Dev. Cell.

[70] Ellies D.L., Church V., Francis-West P., Lumsden A. (2000). The WNT Antagonist CSFRP2 Modulates Programmed Cell Death in the Developing Hindbrain. Development.

