# Supplementary material for: Modeling the Differentiation of Embryonic Limb Chondroprogenitors by Cell Death and Cell Senescence in High Density Micromass Cultures and Their Regulation by FGF Signaling
Source: Cells. 2022 Dec 31;12(1):175. doi: 10.3390/cells12010175 (PMC9818968; doi:10.3390/cells12010175)
Supplement: Supplementary file 1 [file cells-12-00175-s001.zip › cells-2045135-supplementary.pdf]

Table S1: qPCR and MSRE-qPCR chicken specific primers.

qPCR Primers			
Gene	**NCBI Ref. Seq.**	**Fwd Primer**	**Rev Primer**
Ch. Bak1	NM_001030920	ctacgtcaccgaattcatgc	aacattgtccagatcgagtgc
Ch. Bcl2	NM_205339	ttgtacggcaacagtatgagg	ataagcgccaagagtgatgc
Ch. BMP2	NM_204358	tggaatgactggattgttgc	tggaattcaccgaattgacc
Ch. BMP4	NM_205237	agagcctccaggagatcagc	gctgaggttgaagacgaagc
Ch. BMP5	NM_205148	cagcgaaggcactacaagg	gctgctgtcactgcttctcc
Ch. BMP7	XM_417496	aagcacgagctctatgtcagc	cacagtaatacgcagcatagcc
Ch. CTSD	NM_205177	tatggtgagattggcattgg	gatgtctagcaggtgacagtgc
Ch. Dkk1	XM_040702954	cggaactcggcactgacc	tcttggaccagaagtggcg
Ch. DNMT1	NM_206952	cgagttcctgcagaacaacc	gagcgaatcctctgtgaagc
Ch. DNMT3a	NM_001024832	gcaggatagccaagttcagc	cacaggatgtcttccttctcg
Ch. DNMT3b	NM_001024828	caagaggctgaagagcaacc	cgctgttgttcgtaacttcg
Ch. FGFR1	NM_205510	ggtggtgccatcagataagg	ctggtaggtgtggttgatgc
Ch. FGFR2	NM_205319	caagctcctccatgaactcc	ggcagttcatattccgagacc
Ch. FGFR3	NM_205509	ggcggcagaatacttgagg	ctgttggaaggtgcaatcc
Ch. FGFR4	XM_025154976	cgagctgtacatgctgatgc	caggtactcctccgagatgg
Ch. Gapdh	NM_204305	ggtggccatcaatgatcc	gttctcagccttgacagtgc
Ch. GLB1	NM_001278147	tgtcacagcagcattcttagc	acaacaatgtgacggtgtcc
Ch. HDAC2	NM_204831	tgatattgtgcttgccatcc	gcttcttcaacaccatcacc
Ch. HDAC3	NM_204747	tgcctctacgagatggtatcg	gagaatctgcaccacactgc
Ch. HDAC8	XM_004940604	cacaagttttcgccaggatt	gccatcttggataggcacat
Ch. IL6	NM_204628	gcaagaagttcaccgtgtgc	ggcaggttgaggttgttcc
Ch. MMP2	NM_204420	atgatgatgaccgcaagtgg	tcctcggagtgctctaatcc
Ch. MSX1	NM_205488	caagcacaagaccaacagga	tactgcttctggcggaattt
Ch. MSX2	NM_204559	ctctgaggaagcacaagacg	tggacaggtactgcttctgg
Ch. Noggin	NM_204123	ccagcactacctgcacatcc	ggctccttagcaaggtctcg
Ch. Oct4	NM_001110178	ggtatctcgagccattcacc	cttgtgcttgaggtccttgg
Ch. p21	NM_204396	cgtagaccacgagcagatcc	cgtctcggtctcgaagttg
Ch. PRMT5	NM_001320230	gcagtacctggagcatgtgg	actccaggttgtccatcagg
Ch. Scleraxis	NM_204253	caccaacagcgtcaacacc	cgtctcgatcttggacagc
Ch. Sox9	NM_204281	gaggaagtcggtgaagaacg	gatgctggaggatgactgc
Ch. SPRY1	NM_001097524	gccttgcttgctctgttacc	gtgttggagttcttgcatcg
Ch. TET3	XM_015297468	aagaggagaagctgctgtgc	gctcctggtacagtgtgtcg
Ch. Wnt5a	NM_204887	ggtacgaccagttcaagacg	tggtccacgatctctgtgc
**MSRE-qPCR Primers**			
Gene	**CpG Island**	**Fwd Primer**	**Rev Primer**
Ch. Sox9	CpG: 373	gtctctgccggctttacttct	atcaggggaggagaaaacgga
Ch. Sox9	CpG: 373	atttttgcctcggaggagtcg	gagattcatgcgagaaagcgg
Ch. Scleraxis	CpG: 138	ctgtaccccgagatcagcat	ggtgttgacgctgttggtg
Ch. Scleraxis	CpG: 138	acagagcggacgtttggg	cgtatcggtgctaatggggc
